# A Dual-Functional Magnetic Nanoparticle for Rapid Extraction and Targeted Tumor Delivery of Tiancimycin

**DOI:** 10.3390/molecules31193531

**Published:** 2026-10-03

**Authors:** Chengyu Zhang, Peng Zhong, Daxin Zhao, Yun Lv, Ximeng Zhang, Chunyan Chen, Huijuan Yu, Xin Chai, Yunsha Zhang, Jiaxing Zhang, Yuefei Wang, Xiaohui Yan

**Affiliations:** 1 National Key Laboratory of Chinese Medicine Modernization, Tianjin University of Traditional Chinese Medicine, Tianjin 301617, Chinachaix0622@tjutcm.edu.cn (X.C.); 2 Haihe Laboratory of Modern Chinese Medicine, Tianjin 301617, China; 3 School of Integrative Medicine, Tianjin University of Traditional Chinese Medicine, Tianjin 301617, China; 4 Chemical Engineering Research Center, School of Chemical Engineering and Technology, Tianjin University, Tianjin 300072, China; zhangjiaxing7137@tju.edu.cn

**Keywords:** anthraquinone-fused enediynes, tumor-targeting, magnetic nanoparticles, specific separation, triple-negative breast cancer

## Abstract

Anthraquinone-fused enediynes (AFEs) are a class of microbial natural products with remarkable cytotoxicity and exhibit promising application prospects in tumor therapy. Taking tiancimycins (TNMs) as an example, this class of compounds holds great potential against triple-negative breast and liver cancers. However, the low production titers of AFEs in actinobacterial hosts make their enrichment and separation processes both cumbersome and costly. Therefore, the main objective of this study was to develop a rapid and reusable magnetic platform for TNM enrichment and recovery. Inspired by the pi-pi stacking interactions between AFEs and their natural binding proteins in the producing strains, this study designed and prepared alizarin- and folic acid-co-modified magnetic nanoparticles (PNMP-AZ-FA). On the one hand, these nanoparticles can directly and specifically adsorb TNMs from the crude extract of *Streptomyces* sp. CB03234-S, enabling efficient separation. On the other hand, the folic acid modification was explored as a proof-of-concept delivery element for drug-loaded nanoparticles in folate receptor (FR)-expressing tumor models. PNMP-AZ-FA showed loading capacities of 87.8 and 106.7 mg/g for TNM A and TNM D, respectively, and retained high adsorption performance during 20 repeated cycles. Animal experiment results demonstrated that the TNM-loaded nanoparticle (PNMP-AZ-FA@TNM A) improved antitumor activity under the tested conditions, especially in the FR-high KB model; however, receptor-dependent uptake, biodistribution, and pharmacokinetic validation are still required. In summary, this nanoparticle holds great application prospects in the rapid separation and acquisition of AFEs, while its tumor-delivery application should be regarded as preliminary evidence.

## 1. Introduction

Anthraquinone-fused enediynes (AFEs) are some of the most toxic natural products discovered so far [1]. The enediynes can insert into the DNA minor groove to cause DNA double-stranded breaks and cell death at nanomolar levels. They show extreme cytotoxicity against various cancer cell lines and are promising candidates for antitumor drugs [2]. Among the fourteen discovered enediynes, calicheamicin and neocarzinostatin have been successfully translated into anticancer drugs, and C-1027 (lidamycin) is in preclinical studies [3,4,5]. The anthraquinone-fused enediynes (AFEs) are a small group of enediynes featuring an anthraquinone moiety fused to the 10-membered macrocyclic core. Five AFEs (dynemicin A, uncialamycin, tiancimycin A, yangpumicin A, and sealutomicin) were discovered in the past three decades [6]. Tiancimycin A (TNM A) and uncialamycin are currently in preclinical studies [7,8]. The extensive evaluation of AFEs as anticancer drugs requires sufficient supply of these highly toxic compounds. However, due to the complex structures and lability of AFEs, their total synthesis is very challenging and has only been achieved on a small scale to date [9,10]. Microbial fermentation is the main route to produce AFEs in industry. Because of the extreme antibacterial potency of enediynes, the wild-type strains can only produce them in trace amounts (<0.5 mg/L), and large-scale fermentation is needed to provide sufficient compounds for clinical and preclinical applications [11]. *Streptomyces* sp. CB03234 is the original producer of TNM A and TNM D [12]. After fermentation, large quantities of energy and organic solvents are utilized to concentrate and separate the enediynes from the fermentation broth. Therefore, there is an urgent need to find a method to achieve simple, rapid, and specific separation of AFEs from the fermentation broth or crude extracts.

Multiple methods are usually used to extract and separate natural products from plant or microbial crude extracts, for instance, resin adsorption, organic solvent extraction, and membrane separation [13,14,15,16]. These methods are often accompanied by complicated steps and large organic solvent consumption. The development of materials science has led to the synthesis of new materials for medical diagnosis, drug delivery, harmful substance removal, compound separation, and enzyme immobilization [17,18,19,20,21,22]. Magnetic particles hold great potential in compound separation because they are easy to synthesize, concentrate, and reuse [23,24,25,26]. CoNi-doped magnetic nanoparticle-derived CoNi-LDH hollow nanocages can safely remove bisphenol A from foods like bottled water, milk, honey, and eggs [27]. Polystyrene sulfonate-functionalized magnetic nanoparticles (PSS@SMPs) were synthesized for the rapid extraction of calcium-containing nutrients [28,29]. In *Streptomyces* sp. CB03234, the self-resistance proteins (TnmS1–S3) sequester TNMs within their cavity. Structural analysis reveals that the anthraquinone moiety of TNMs is sandwiched between two aromatic tryptophan residues of the binding proteins via electrostatic interactions, separating the reactive enediyne from chromosomal DNA and protecting the host from TNM toxicity [30]. By emulating this mechanism to design a material capable of specific AFE capture and rapid extraction, a fast, convenient, and cost-effective process can be achieved, thereby substantially lowering the application cost of AFEs and facilitating their broader utilization.

Although AFEs exhibit significant cytotoxic effects against tumors, their high toxicity also leads to severe systemic side effects. Therefore, the current research focus of AFE-based therapeutic strategies lies in selecting tumor-targeting carriers to achieve effective loading of AFEs, thereby reducing their toxicity through targeted therapy. The FR has emerged as a highly attractive target for tumor-selective drug delivery in tumor-targeted therapy, as it is highly expressed in a range of common malignancies--such as breast, ovarian, and non-small cell lung cancer--while remaining at extremely low levels in normal tissues [31,32]. As a natural ligand for FR, folic acid (FA) offers the advantages of high affinity, low toxicity, and small molecular size [33]. Its water solubility and ease of modification for integration into nanocarriers provide distinct advantages for the construction of efficient targeted delivery systems [34]. In the present study, FA modification was therefore used as a preliminary delivery-design element, whereas the main validated objective was to establish an AZ-containing magnetic platform for rapid TNM capture and recovery.

Inspired by the TnmS-AFEs sequestration mechanism, this study designed a magnetic nanoparticle modified with anthraquinone molecules. The selected anthraquinone molecule is alizarin (AZ), which is derived from the traditional Chinese medicinal herb Rubia cordifolia and is a pharmacologically active anthraquinone compound [35]. The planar structure of AZ enables the material to specifically adsorb AFEs from complex matrices. Controls confirmed AZ-specific capture: bare particles gave ~8–9% loading vs. ~99% for PNMP-AZ-FA (Table S4), ruling out nonspecific adsorption. The tumor-targeting nanocarrier was sequentially modified with polyethylenimine (PEI), followed by AZ and FA, enabling AFE capture and exploratory folate-associated delivery. In this study, we selected TNM A, a type of AFE with high antitumor activity, for loading and targeted therapy experiments. Animal experimental results confirmed its favorable antitumor effect. These biological results should be interpreted as preliminary evidence of improved antitumor activity rather than definitive proof of receptor-dependent uptake or tumor accumulation.

## 2. Results and Discussion

### 2.1. Molecular Docking Analysis

**Figure 1:**
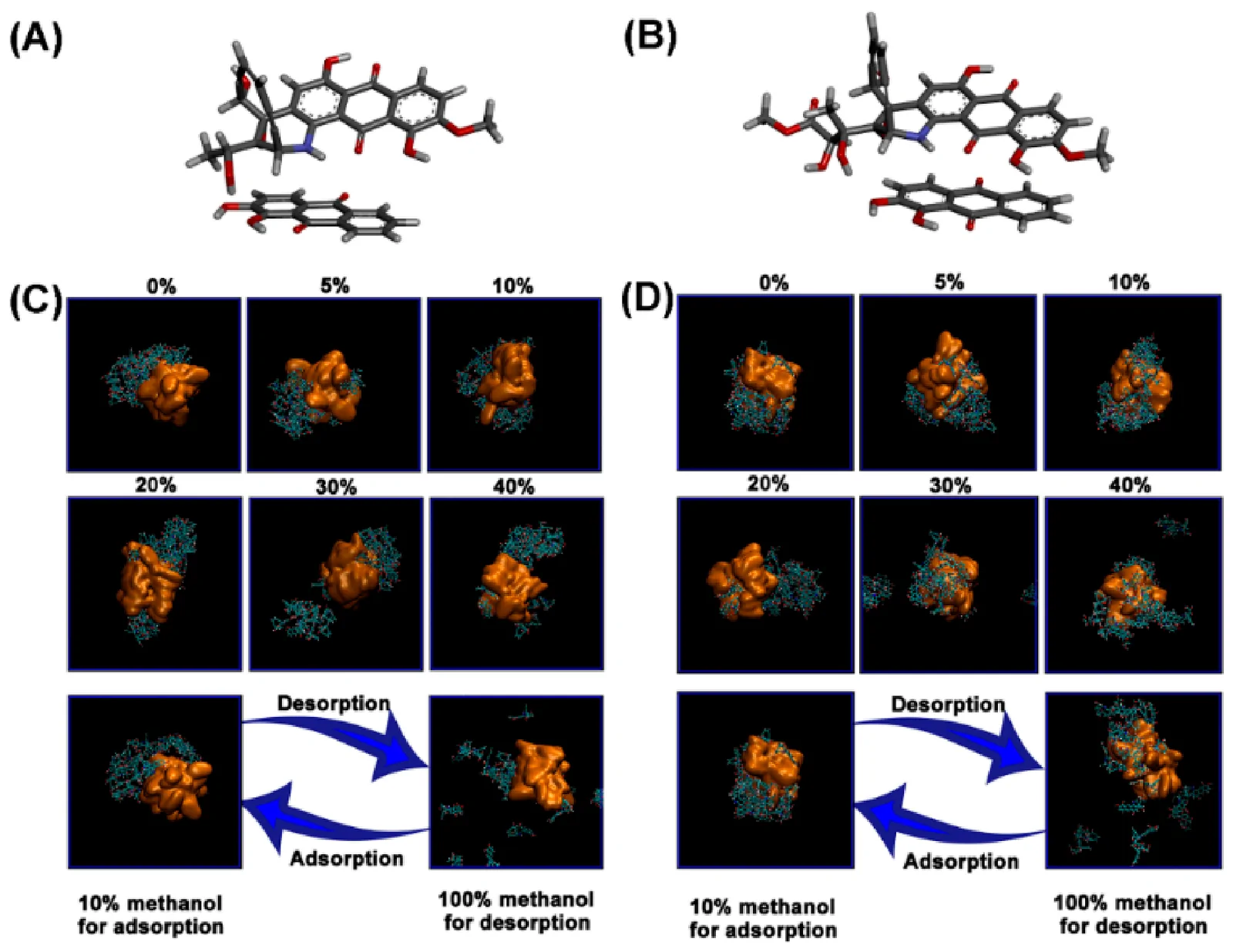
Molecular docking and molecular dynamics simulation. (**A**) AZ/TNM A molecular docking results; (**B**) AZ/TNM D molecular docking results; the adsorption/desorption performance for (**C**) PEI-AZ/TNM A and (**D**) PEI/TNM D calculated by molecular dynamics simulation in different concentrations of methanol/water spheres; 0%, 5%, 10%, 20%, 30%, 40%, and 100% represent different methanol concentrations in the spheres.

To investigate the theoretical feasibility of adsorbing TNMs by AZ-modified NPs, molecular docking and quantum chemical calculations were performed using TNM A and TNM D as models. The anthraquinone moieties of the TNM A (or TNM D) molecule exhibited a parallel structure with AZ, and the short distance between them indicated the formation of pi-pi interactions (Figure 1A,B). The binding energies of AZ/TNM A and AZ/TNM D were −71.765 and −88.113 kJ, respectively (Table S1), indicating a more stable structure of the complex. These results theoretically verified the feasibility of using AZ to adsorb TNMs. However, because the adsorption experiments mainly used the complete PNMP-AZ-FA material, additional AZ-free particle controls, such as PNMP or PNMP-FA, would be required to fully exclude nonspecific adsorption to the particle surface. In aqueous solution, AZ molecules connected to PEI may interact with each other in space due to their proximity, thus affecting the adsorption of TNMs. Considering this, a monomer structure of PEI was used to build a PEI-AZ complex model (Figure S1).

TNM A or TNM D was used to calculate the spatial interaction with PEI-AZ. Because the hydrophobic components may interfere with TNM adsorption, we proposed that a low fraction of organic solvents could promote the dispersion of TNMs and that high solvent concentrations may affect the interaction between TNMs and AZ on the NP surface. Therefore, different ratios of methanol were incorporated into the kinetic simulation system of the interaction between PEI-AZ and TNM A/D to improve the dispersion, ensure the adsorption of TNMs, and reduce the interference. According to the dynamics simulation results (Figure 1 and Figures S2–S4), at relatively low methanol concentrations (0%, 5%, and 10%, *v*/*v*), TNM A and TNM D could be tightly adsorbed onto the surface of PEI-AZ. The adsorption rates in 5% and 10% methanol were faster than those in pure water, demonstrating that methanol promotes the dispersion of TNMs and enhances the capture of TNMs (Figures S2 and S3). As the methanol concentration increased from 20% to 40%, the adsorption of PEI-AZ for TNM A and TNM D was gradually weakened, and the release of TNM A or TNM D increased. These results indicated that 5% and 10% methanol might be the optimal conditions to extract the TNMs.

### 2.2. Characterization of the Synthesized NPs

**Figure 2:**
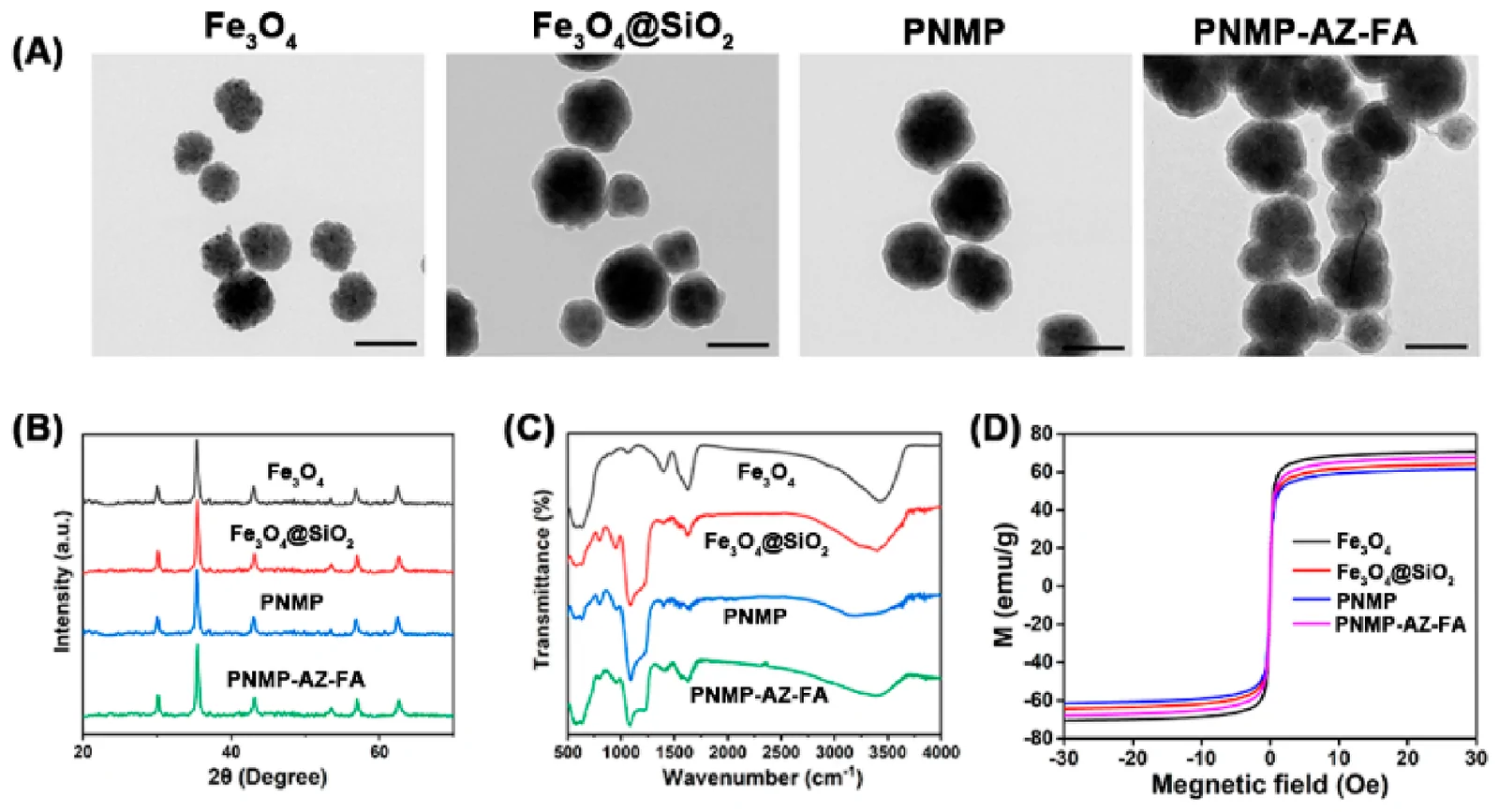
Characterization of the synthesized nanoparticles. (**A**) TEM image; (**B**) XRD patterns; (**C**) FTIR spectra; (**D**) VSM features of Fe_3_O_4_, Fe_3_O_4_@SiO_2_, PNMP, and PNMP-AZ-FA. The scale bar was 200 nm.

To obtain PNMP-AZ-FA, AZ was first modified with epoxy groups to form AZ-ECH. After the synthesis and purification, the high-resolution mass spectrum and 1H-NMR analysis confirmed that AZ-ECH was obtained (Table S3). The synthesized Fe_3_O_4_, Fe_3_O_4_@SiO_2_, Fe_3_O_4_@SiO_2_@PEI (shortened as PNMP), and PNMP-AZ-FA were characterized using TEM, XRD, Fourier transform infrared spectroscopy (FTIR), and vibrating sample magnetometry (VSM). The synthesized NPs showed a regular spherical shape, with an average particle diameter of around 200 nm (Figure 2A). The surfaces of Fe_3_O_4_@SiO_2_, PNMP, and PNMP-AZ-FA showed easily identifiable coating layers, indicating the coverage of SiO_2_ and the modification of PEI/AZ on the surface of the NPs. The XRD patterns of Fe_3_O_4_@SiO_2_, Fe_3_O_4_@SiO_2_@PEI, and PNMP-AZ-FA were similar to the Fe_3_O_4_ sample. The peaks at 2θ = 30.4, 35.2, 43.5, 53.9, 57.5, and 62.3 degrees are characteristic of face-centered cubic magnetite Fe_3_O_4_ [36,37,38], indicating the formation of Fe_3_O_4_ crystals (Figure 2B). For FTIR data (Figure 2C), the characteristic bands at 587 and 3400 cm^−1^ are the Fe-O and O-H stretching vibration absorption peaks of these NPs. The band at 630 cm^−1^ and the broad and intense band at 1085 cm^−1^ in the Fe_3_O_4_@SiO_2_ sample correspond to the Si-O-Si bonds, and the band at 1250 cm^−1^ is the absorption peak of C-N in the PEI molecule of PEI-modified NPs. Moreover, the prominent vibrating sample magnetometry (VSM) values (Figure 2D) of Fe_3_O_4_, Fe_3_O_4_@SiO_2_, Fe_3_O_4_@SiO_2_@PEI, and PNMP-AZ-FA indicated that the synthesized NPs could be quickly separated from the solution via a magnet.

The physicochemical properties of Fe_3_O_4_, Fe_3_O_4_@SiO_2_, and PNMP-AZ-FA are summarized in Table S4. The zeta potential decreased progressively from −16.7 ± 1.3 mV for Fe_3_O_4_ to −27.8 ± 2.1 mV for Fe_3_O_4_@SiO_2_ and further to −35.3 ± 2.9 mV for PNMP-AZ-FA, indicating a stepwise increase in surface negative charge density after silica coating and subsequent polymer/ligand modification. Concurrently, the average hydrodynamic diameter gradually increased from 156.3 ± 17.3 nm (Fe_3_O_4_) to 184.5 ± 21.5 nm (Fe_3_O_4_@SiO_2_) and 202.3 ± 26.7 nm (PNMP-AZ-FA), further validating the effectiveness of the layer-by-layer modification strategy. Notably, the loading efficiencies of both TNM A and TNM D were relatively low (approximately 9%) for the unmodified and silica-coated particles, whereas those for PNMP-AZ-FA dramatically increased to 98.9 ± 2.8% and 99.5 ± 1.1%, respectively. This substantial improvement demonstrates that the PNMP-AZ-FA material possesses an exceptionally high loading capacity for TNM A and TNM D.

### 2.3. Specificity of TNM Adsorption

**Figure 3:**
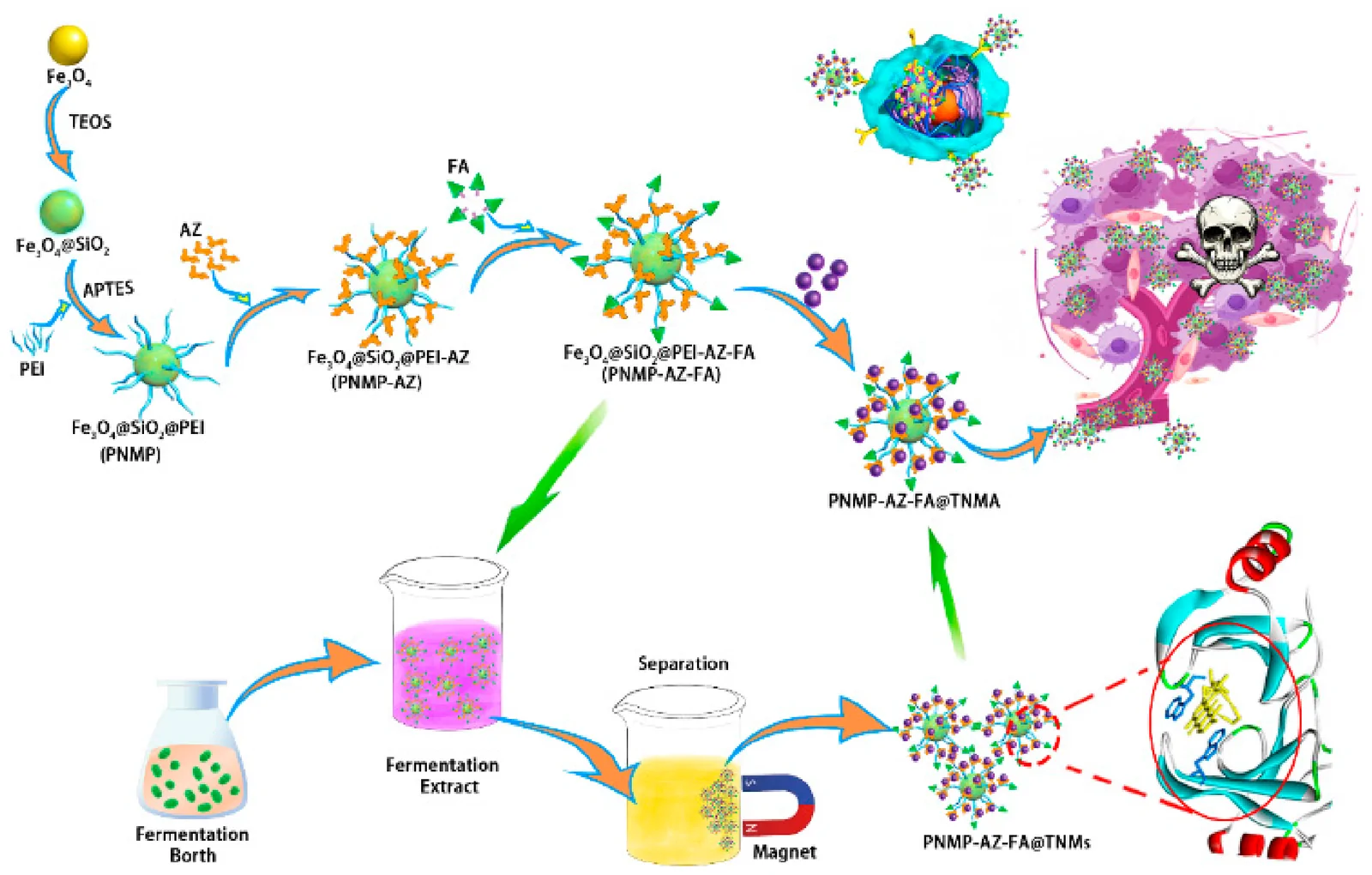
Scheme for synthesis of PNMP-AZ-FA and its application in the specific separation of TNMs.

**Figure 4:**
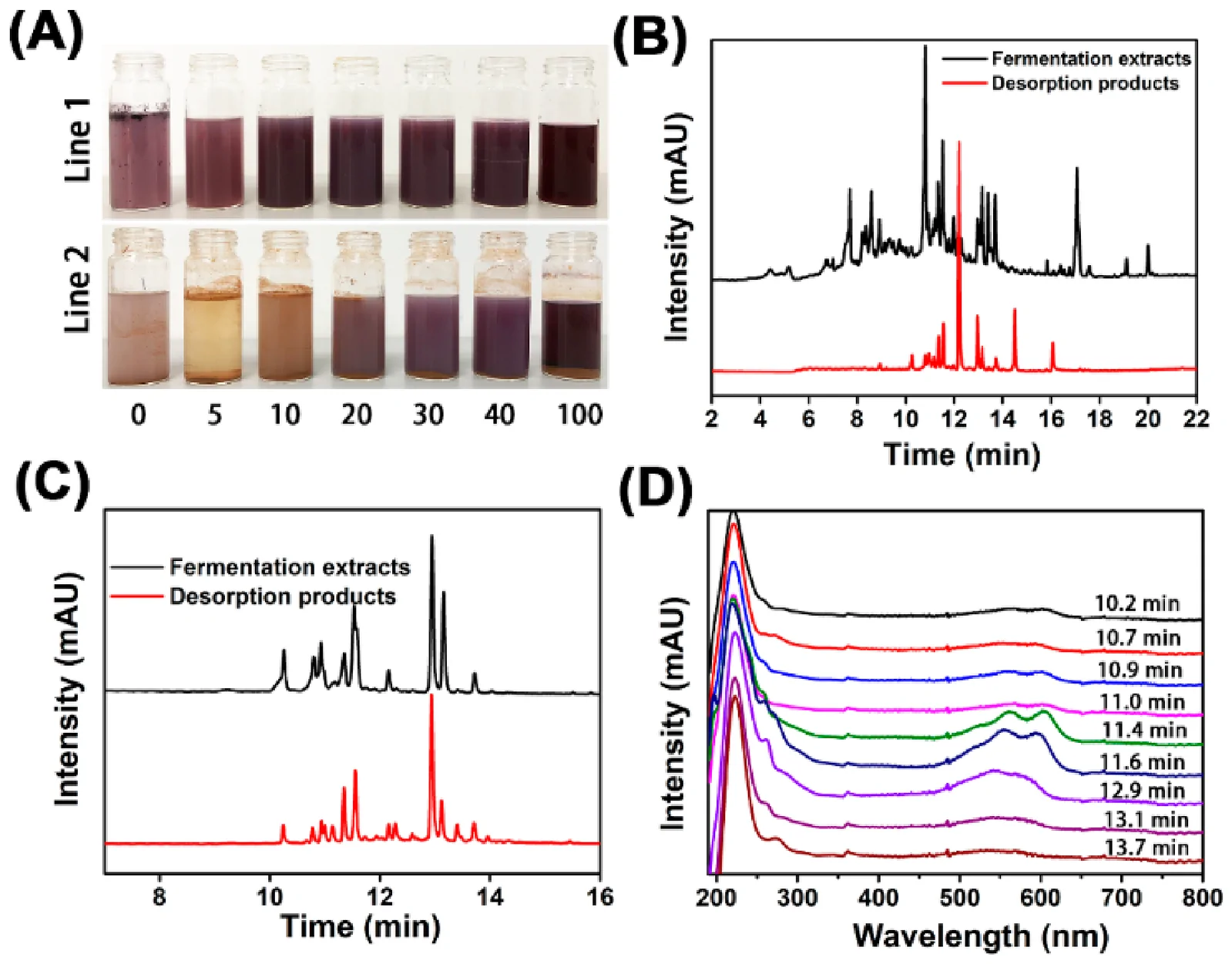
Adsorption and HPLC analysis of the performance of PNMP-AZ-FA. (**A**) Adsorption of TNMs in different concentrations of methanol; 0, 5, 10, 20, 30, 40, and 100 represent the concentrations of methanol in the adsorption system. Lines 1 and 2 represent the PNMP-AZ-FA particles before and after adsorption via a magnet, respectively. HPLC analysis of the fermentation extracts and the substances adsorbed by PNMP-AZ-FA in 10% methanol at (**B**) 254 nm and (**C**) 540 nm. (**D**) Full wavelength scan of HPLC-detected elements of the obtained products in 10% methanol.

After characterization, the synthesized PNMP-AZ-FA was used to extract TNMs. The synthesis and application of PNMP-AZ-FA in the specific isolation of TNMs are illustrated in Figure 3. Firstly, the Fe_3_O_4_ particles were synthesized via a hydrothermal method. The SiO_2_ layer was modified on the surface of Fe_3_O_4_ using TEOS and APTES, and the amino group was introduced to ensure the subsequent grafting reaction. After that, Fe_3_O_4_@SiO_2_ was encapsulated with PEI (a water-soluble polymer that can improve water solubility and provide more amino groups). Finally, the reaction between epoxy and the amino group was carried out to link the AZ onto the amino group of PEI, giving the nanoparticles the ability to capture TNMs. The crude extract of CB03234-S contains various substances, which increase the difficulty of extracting and separating the TNMs. To eliminate nonspecific binding of substances other than the TNMs and realize specific adsorption of the AFEs, different concentrations of methanol were added to the suspension. As shown in Figure 4A, the crude extract was not well dispersed in pure water, and the lipid layer floated on the surface of the water. The solubility improved as the concentration of methanol increased from 0% to 5% and 10%. More TNMs were adsorbed on the surface of PNMP-AZ-FA in 5% methanol than in 10% methanol. Only a small amount of the purple compounds remained in the solution, indicating that most of the purple TNMs were adsorbed by the magnetic material. However, the sticky substance attached to the surface of the recovered magnetic particles showed that many impurities were also adsorbed. Utilizing 10% methanol could thoroughly disperse the components of the crude extract as a solution rather than a viscous emulsion. With further increase in methanol concentration, the adsorption of TNMs on the surface of magnetic particles gradually decreased, which is in line with the kinetic simulation results. These results demonstrate that PNMP-AZ-FA can effectively capture the TNMs from the suspension, and 10% methanol is suitable for the adsorption of TNMs by this material.

To ensure that the compounds captured by PNMP-AZ-FA were TNMs, the NPs were eluted with methanol and subjected to HPLC and HPLC-MS analysis. Figure 4B,C show the HPLC profiles of the total crude extract and the compounds adsorbed by PNMP-AZ-FA. According to our previous research [30,39,40,41], the TNMs have a characteristic absorption at 540 nm, while other substances from CB03234-S have no absorption at this wavelength. Only a few compounds were adsorbed by PNMP-AZ-FA, and analysis of the UV spectra of the captured samples revealed that they all had a specific absorption at 540 nm, indicating the specific adsorption of TNMs by the PNMP-AZ-FA NPs (Figure 4D, Figures S5 and S6). The two compounds with the retention times of 12.9 min and 13.1 min in HPLC were TNM A and TNM D, respectively (Figure S7).

### 2.4. Operability, Loading Capacity of PNMP-AZ-FA, and Structural Stability of Adsorbed Substances

**Figure 5:**
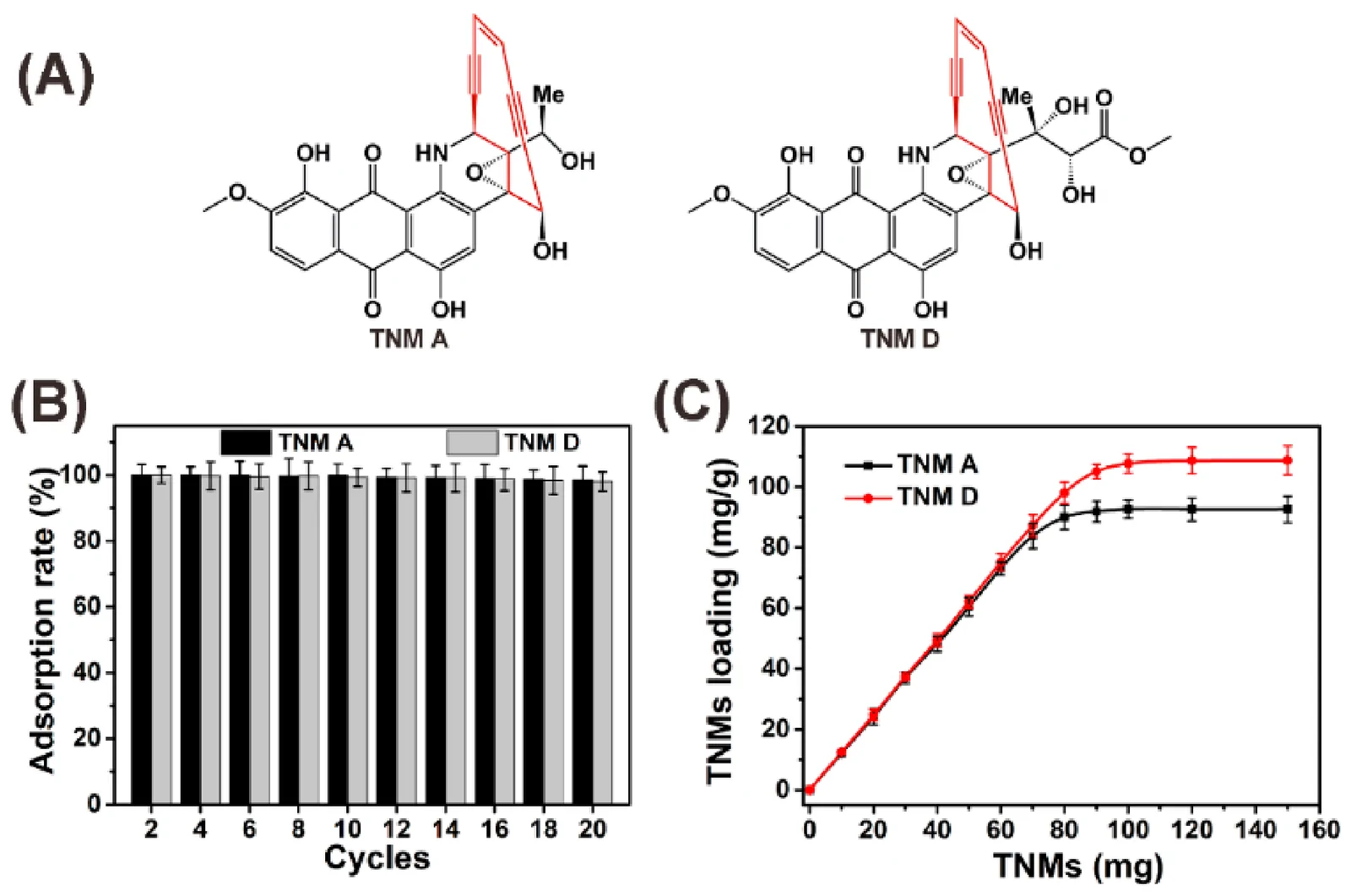
(**A**) The structures of TNM A and TNM D. (**B**) The recycling performance of PNMP-AZ-FA for the adsorption of TNM A and TNM D in 10% methanol. (**C**) Loading capacities of TNM A and TNM D onto PNMP-AZ-FA.

The operability and capacity of PNMP-AZ-FA were determined using TNM A and TNM D (Figure 5A). After twenty cycles of repeated adsorption–desorption operations, the cycle recovery (defined in Section 2.6 as the ratio of eluted TNMs to the initial added amount) of PNMP-AZ-FA for both TNM A and TNM D remained above 95% (Figure 5B; mean ± SD, *n* = 3), indicating good operational stability of the material. The adsorption capacity of TNM A and TNM D was also tested to evaluate the loading capacity of PNMP-AZ-FA for the TNMs. The loading capacities of this material for TNM A and TNM D were 87.8 and 106.7 mg/g (Figure 5C). The different loading capacities for TNM A and TNM D are attributed to their different molecular weights (MWs). TNM A and TNM D have molecular formulas of C_27_H_19_NO_8_ (MW 485.11) and C_30_H_23_NO_11_ (MW 573.51), respectively. The molar loading capacities of the PNMP-AZ-FA NPs for TNM A and TNM D were equal (0.19 mM per gram of NPs).

The TNMs are highly reactive and can undergo cycloaromatization in acidic and reductive environments [42]. Therefore, it is crucial that the adsorbent neither reacts with the compounds to be adsorbed nor causes cyclization of the enediynes during the extraction process. The reliability of the PNMP-AZ-FA NPs for separating the AFEs was evaluated using TNM A and TNM D as the ligands. The PNMP-AZ-FA NPs loaded with TNM A or TNM D were stored at room temperature for 12 h and then desorbed with methanol. The status of TNM A or TNM D before and after adsorption by PNMP-AZ-FA was analyzed, and the results are shown in Figure S7a,b. HPLC results showed that there was no difference between the desorbed TNM A (or TNM D) and the standard TNM A or TNM D (samples before adsorption by PNMP-AZ-FA, 13.1 min for TNM A and 12.9 min for TNM D, respectively). The HPLC-MS results showed the same molecular weights as those of the samples before adsorption by PNMP-AZ-FA (Figure S7c,d, MW 485 for TNM A and 573 for TNM D). The above results indicate that PNMP-AZ-FA can maintain the structural stability of TNMs during the specific adsorption procedure.

### 2.5. Comparison of Traditional and the PNMP-AZ-FA Extraction Methods

**Figure 6:**
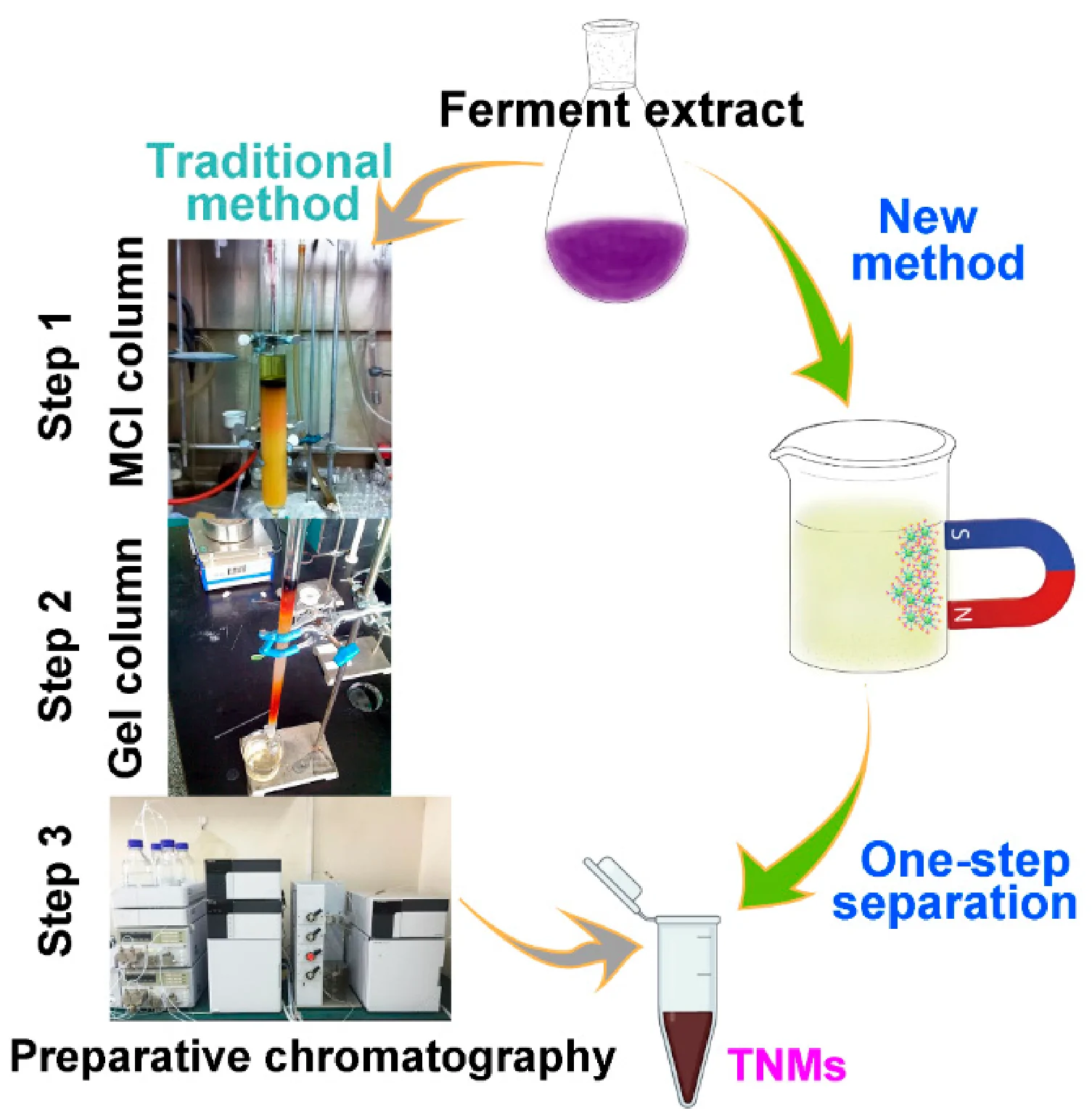
TNM separation steps by the traditional method and the PNMP-AZ-FA method.

Many microbial secondary metabolites are usually biosynthesized with low yields by their producers. To obtain sufficient compounds for biosynthetic and clinical studies, large-scale fermentation is often required, thus causing problems in concentrating the fermentation products and separating the target compounds from other metabolites. To separate the TNMs, the traditional extraction process includes multiple steps, including extraction with ethyl acetate, rough separation via silica gel- and MCI resin-based column chromatography (CC), size exclusion chromatography with Sephadex LH-20, and further separation by preparative HPLC 12. Factors such as cumbersome steps, large organic solvent consumption, and dependence on specialized equipment contribute to the high cost for the purification of target natural products. Compared with the traditional process, the PNMP-AZ-FA NPs can effectively separate the TNMs from other metabolites in crude extract in a rapid, facile, and solvent-saving manner (Figure 6). We therefore describe the comparison as a direct reagent-cost estimate rather than a complete process-cost comparison. Traditional methods separate TNMs by using MCI and Sephadex LH-20 gels, with MeOH and EtOAc as extraction reagents (Table S2). To separate TNMs from the crude extract (7.5 L fermentation) via the traditional method, 5 L of MeOH, 6 L of EtOAc, 50 g of MCI gel, and other reagents were consumed, making the single cost reach 432.4$. However, for the PNMP-AZ-FA method, the cost was only 2.1$. This estimate excludes labor, equipment depreciation, nanoparticle synthesis/regeneration beyond the listed reagents, final-yield normalization, final-purity normalization, and later preparative HPLC purification. Good reusability, the ease of synthesis and operation, and the low reagent consumption properties make the PNMP-AZ-FA NPs a promising matrix for the specific separation of natural products with an anthraquinone moiety.

### 2.6. FR Expression Analysis and In Vitro Antitumor Evaluation in Cultured Cells

**Figure 7:**
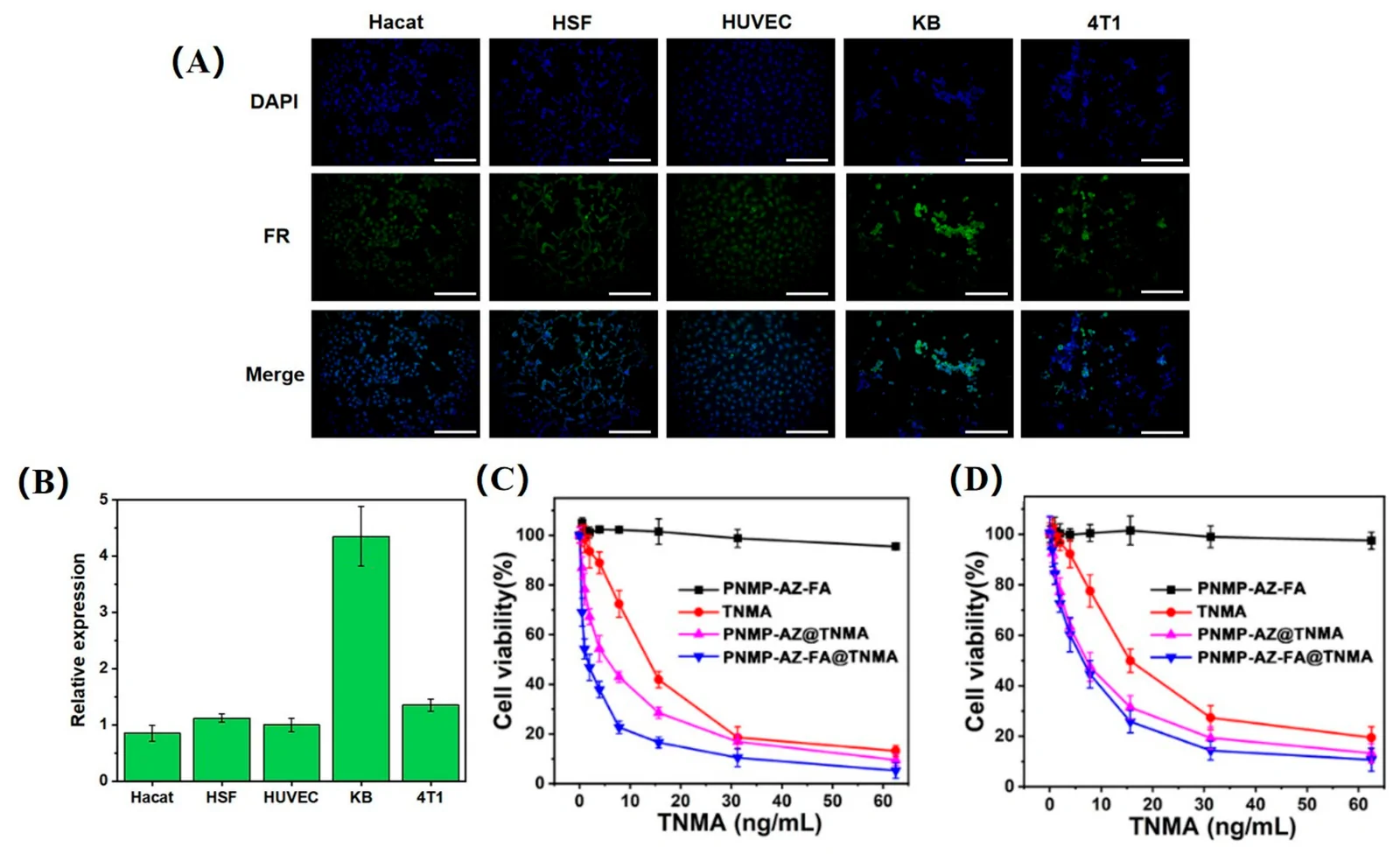
Expression of FR in different cell lines and the antitumor efficacy of drugs. (**A**) Immunofluorescence analysis of FR expression in different cell lines, and (**B**) quantitative analysis results. Evaluation of the antitumor efficacy of different materials against (**C**) KB and (**D**) 4T1 cells.The scale bar was 100 um.

Using immunofluorescence analysis, the expression status of FR in cells from different sources was examined. The results showed that FR-related fluorescence signals were weak in several normal human cell types, including epidermal cells (HaCaT), fibroblasts (HSF), and vascular endothelial cells (HUVEC). FR-related fluorescence signals were also relatively low in triple-negative breast cancer cells (4T1). However, in oral epidermoid carcinoma cells (KB), the FR-related green fluorescence intensity was significantly enhanced, indicating aberrantly high expression of FR in KB cells (Figure 7A,B).

The inhibitory effects of different concentrations of PNMP-AZ-FA, TNM A, PNMP-AZ@TNM A, and PNMP-AZ-FA@TNM A on tumor cell proliferation were further evaluated using the MTT assay. In FR-high-expressing KB cells, the PNMP-AZ-FA@TNM A group exhibited significantly higher antitumor activity compared to the other treatment groups, and the inhibitory effect was enhanced with increasing concentrations of TNM A (Figure 7C). In FR-low-expressing 4T1 cells, the PNMP-AZ-FA@TNM A group also demonstrated the strongest inhibitory effect, with activity increasing as TNM A concentration increased (Figure 7D). However, the inhibition rates under equivalent treatment conditions were significantly lower than those observed in FR-high-expressing KB cells. In H9C2 cells with even lower FR expression, the PNMP-AZ-FA@TNM A group and the PNMP-AZ@TNM A group exhibited comparable inhibition rates (Figure S8). These findings are consistent with an FR-associated effect of FA modification; however, folate-competition, receptor-blocking, direct uptake comparison, and biodistribution experiments were not performed, so the data should not be interpreted as definitive proof of receptor-dependent uptake.

### 2.7. In Vivo Antitumor Testing

**Figure 8:**
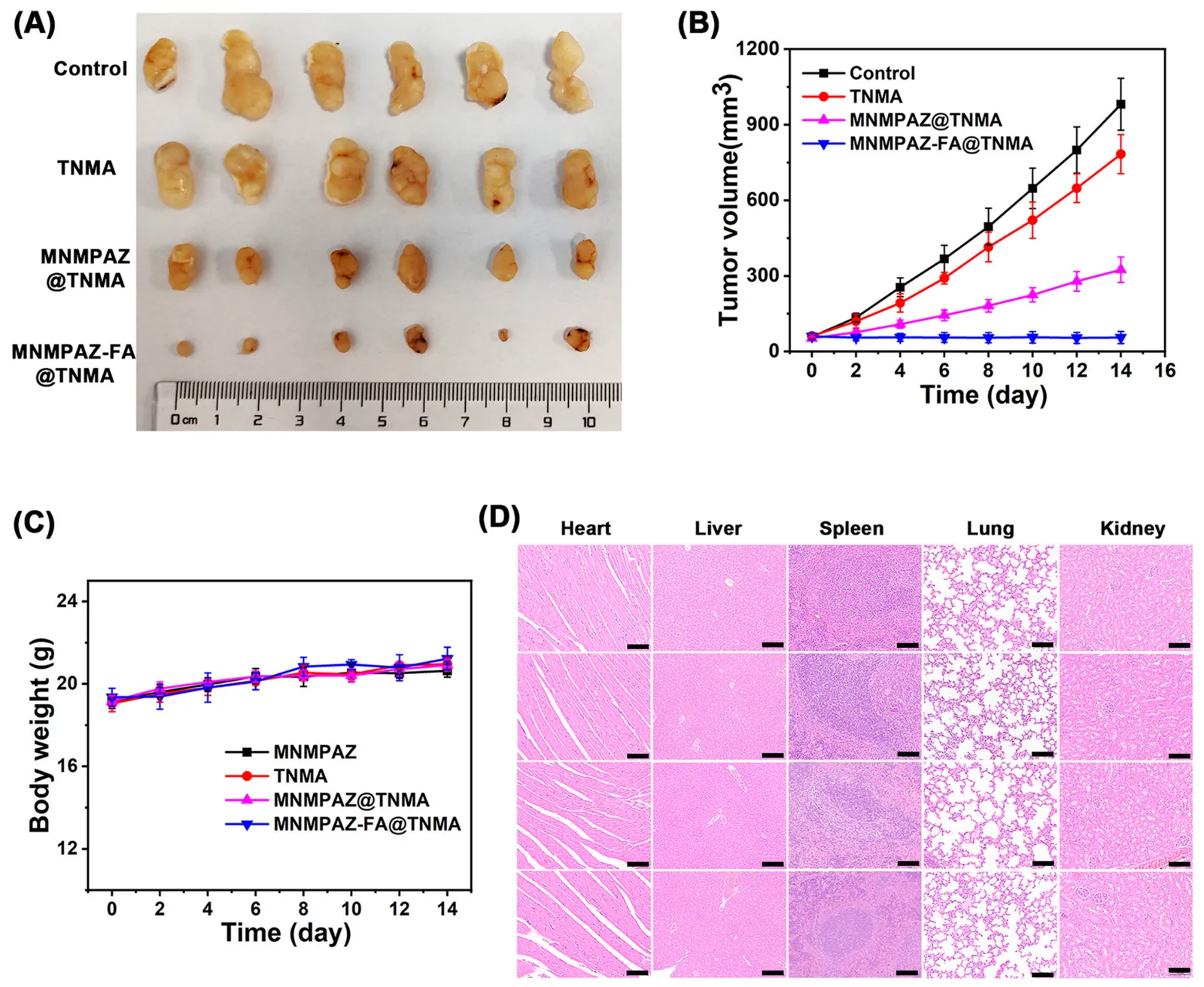
PNMP-AZ-FA@TNM A exerts effective antitumor effects in vivo. (**A**) Representative images of harvested tumors from each treatment group (*n* = 6). (**B**) Tumor growth curves over the treatment period. (**C**) Body weight changes of mice during treatment. (**D**) Histopathological analysis of major visceral organs (H&E staining).The scale bar was 100 um.

To evaluate the in vivo antitumor efficacy of the nanoparticles, nude mice bearing KB tumors were randomly divided into four groups and received corresponding treatments (Figure 8A and Figure S9). Body weight changes of mice in each group were monitored regularly throughout the treatment period (Figure 8C). After the final administration, tumor tissues were excised for photographic analysis (Figure 8A and Figure S9) and tumor-volume evaluation. Results showed that the PNMP-AZ-FA@TNM A group exhibited the smallest tumor volumes with an isolated growth pattern, and tumor volumes were significantly lower than those in the control group (*p* < 0.05). During the treatment period, tumors in the control group grew rapidly. In contrast, the tumor volume in the PNMP-AZ-FA@TNM A group decreased, indicating that this formulation significantly suppressed tumor progression. The PNMP-AZ@TNM A group showed considerably enhanced antitumor activity compared to the free TNM A group, but was still inferior to the PNMP-AZ-FA@TNM A group. This difference is consistent with a contribution from FA modification in the KB model, but direct tumor accumulation and receptor-dependent uptake were not measured.

To further explore whether the therapeutic difference was associated with FR expression, a 4T1 tumor-bearing mouse model with low FR expression was established and evaluated. The results showed no significant difference in antitumor efficacy between the PNMP-AZ@TNM A and PNMP-AZ-FA@TNM A groups in this model, which is consistent with an FR-associated contribution but does not by itself confirm receptor-dependent targeting (Figure S10). Receptor-blocking, uptake, biodistribution, pharmacokinetic, hematological, and biochemical studies are still needed to confirm the targeting mechanism and systemic safety profile.

## 3. Materials and Methods

### 3.1. Materials

FeCl_3_·6H_2_O, sodium citrate (NaCA), sodium acetate (NaAc), ethylene glycol (EG), NaCl, and NH_3_·H_2_O (25%, *w*/*v*) were purchased from Adamas-beta (Beijing, China). Hydrochloric acid, calcium carbonate, methanol, ethanol, tetraethyl orthosilicate (TEOS), alizarin (AZ), 3-aminopropyl triethoxysilane (APTES), benzyl triethylammonium chloride (TEBAC), glutaraldehyde (GA), copper sulfate pentahydrate, anhydrous sodium iodide, epichlorohydrin, trifluoroacetic acid (TFA), and polyethyleneimine (PEI, average degree of polymerization 600) were supplied by Aladdin (Shanghai, China). The ISP medium 4 (ISP4), tryptic soy broth (TSB), soluble starch, macroporous resin HP20 (HP2-MGL), and yeast extract were obtained from Sangon Biotech (Shanghai, China). *Streptomyces* sp. CB03234-S is a high TNM producer kept in our laboratory.

### 3.2. Molecular Dynamics and Quantum Chemistry Simulation

The molecular structures of AZ, TNM A, and TNM D were constructed using the Gaussian View 6 (Gaussian, Inc., Wallingford, CT, USA) program. DFT calculations were performed using the Gaussian 16 (Gaussian, Inc., Wallingford, CT, USA) software package. The B3LYP functional and 6–31G* basis set were employed to calculate the single-point energy of both monomers and dimers of the AZ/TNM A and AZ/TNM D complexes. Thus, the interaction energies of AZ/TNM A and AZ/TNM D were obtained. The 3D structure of PEI-AZ was modified by Discovery Studio Visualizer 2019 (Dassault Systèmes BIOVIA, San Diego, CA, USA). Molecular docking of PEI-AZ/TNM A or PEI-AZ/TNM D was performed with AutoDock Vina v1.2.7 at different methanol/water ratios (the molar ratios of methanol/water were 1/42, 1/20, 1/9, 1/5, 3/10, 10/0, which represent methanol concentrations of 5%, 10%, 20%, 30%, 40%, 100%, respectively) [43].

### 3.3. Strain Culture, Fermentation, and Extraction

Firstly, *Streptomyces* sp. CB03234-S was cultured on a fresh ISP4 agar plate at 28 °C for 10 days. The spores were inoculated into fifteen flasks (250 mL) containing 50 mL of TSB medium and cultured at 28 °C and 220 rpm for 36 h as the seed. Then the seed was inoculated into 15 two-liter flasks, each containing 500 mL of production medium (30 g/L soluble starch, 5 g/L yeast extract, 2 g/L CaCO_3_, 0.1 g/L CuSO_4_·5H_2_O, 0.005 g/L NaI, 10 g/L HP2-MGL, pH 7.3) at 28 °C and 220 rpm for 7 days. After the fermentation, the pellets and the HP2-MGL resin from the 7.5-L fermentation were collected by centrifugation (4000 rpm, 30 min). The precipitate was air-dried for two days and then extracted with methanol five times (500 mL each time). The insoluble solid was removed using filter paper, and methanol was evaporated with a rotary evaporator. The crude extract was stored for further study.

### 3.4. Preparation of Fe_3_O_4_, Fe_3_O_4_@SiO_2_, and PNMP

The Fe_3_O_4_ core was synthesized via the modified Stöber method [44]. Briefly, 1.3 g of FeCl_3_∙6H_2_O and 0.4 g of Na_3_Cit·2H_2_O were mixed with 80 mL of EG. After stirring for 20 min, 2.4 g of NaAc was added to the FeCl_3_-EG system and stirred for another 20 min. The dark-yellow solution was transferred into a polytetrafluoroethylene-lined autoclave and heated at 200 °C for 8 h. The obtained Fe_3_O_4_ superparamagnetic NPs were collected via a magnet, followed by washing with deionized water and ethanol three times, respectively. The Fe_3_O_4_ magnetic powder was dried at 60 °C in a drying oven and stored at room temperature for further studies.

To prepare Fe_3_O_4_@SiO_2_, 0.8 g of dried Fe_3_O_4_ NPs were added to 400 mL of diluted HCl (20 mM) and sonicated for 15 min to remove the impurities on the surface. After washing with deionized water three times, 640 mL of ethanol, 160 mL of deionized water, and the Fe_3_O_4_ NPs were added to a round-bottom flask. The mixture was sonicated for 15 min to disperse the NPs completely. Then, 8 mL of NH_3_·H_2_O was slowly added to the flask, and the mixture was stirred for 5 min to keep the surface of the Fe_3_O_4_ NPs in an alkaline environment. TEOS (4.8 mL) was added dropwise to the mixture to start the reaction. After stirring for 4 h, the Fe_3_O_4_@SiO_2_ NPs were collected and washed with deionized water three times, followed by drying thoroughly in the oven (60 °C). Afterwards, 0.4 g of dried Fe_3_O_4_@SiO_2_ powder was added to 600 mL of ethanol and sonicated for 20 min to disperse the NPs. Then 40 mL of NH_3_·H_2_O and 4.5 mL of APTES were added to the mixture, followed by stirring with a mechanical agitator for 2 h. The APTES-modified Fe_3_O_4_@SiO_2_ was collected and dried after washing first with deionized water three times and then with ethanol three times.

To synthesize the Fe_3_O_4_@SiO_2_@PEI (short for PNMP), 0.4 g of APTES-modified Fe_3_O_4_@SiO_2_ NPs were dispersed into 400 mL of PBS buffer (pH 8.0, 50 mM) containing 0.5% (*w*/*v*) of GA and stirred at room temperature for 2 h. The GA-modified Fe_3_O_4_@SiO_2_ particles (Fe_3_O_4_@SiO_2_-GA) were separated and washed with deionized water three times. Then PEI was grafted onto the surface of the Fe_3_O_4_@SiO_2_ by adding the Fe_3_O_4_@SiO_2_-GA NPs into 400 mL of freshly prepared PBS buffer containing 0.1% (*w*/*v*) PEI, followed by stirring for 1 h. Subsequently, the PEI-modified magnetic NPs (PNMP) were collected via an external magnet, followed by washing first with deionized water three times and then with ethanol three times. The obtained materials were dried for 8 h at 60 °C in the oven.

### 3.5. Synthesis of PNMP-AZ-FA

To synthesize PNMP-AZ-FA, PNMP (0.1 g) was dispersed in 50 mL of Tris-HCl buffer (50 mM, pH 8.0) and then mixed with 1 mL of DMSO containing 60 mg of AZ-ECH (obtained by the preparation method described in our previous study [45]), followed by stirring at 70 °C for 20 h. Afterward, the NPs were collected using a magnet and washed with methanol several times until the free AZ-ECH was removed completely. The PNMP@AZ was then separated using a magnetic separation method and dried at 60 °C for 8 h. PNMP-AZ-FA was prepared via EDC/NHS-mediated amidation. Briefly, 0.05 g of folic acid (FA) was dissolved in MES buffer (0.1 M, pH 5.5), followed by sequential addition of EDC·HCl and NHS to activate the carboxyl groups. After stirring at room temperature for 30 min, 0.2 g of PNMP@AZ nanoparticles was added, and the reaction proceeded for another 2 h. The resulting PNMP-AZ-FA nanoparticles were magnetically separated, washed three times with PBS (pH 7.4) and deionized water to remove unreacted reagents, and finally lyophilized for storage.

### 3.6. Adsorption of TNMs and Recycling of PNMP-AZ-FA

TNM adsorption was carried out in a water/methanol dispersion. Firstly, 0.4 g of PNMP-AZ-FA was added to 450 mL of deionized water, followed by sonication for 20 min to completely disperse the NPs. Moreover, 1 g of crude extract of CB03234-S prepared above (equivalent to the extract from 2 L of bacterial fermentation product) was dissolved in 50 mL of methanol and mixed with 450 mL of PNMP-AZ-FA dispersion to adsorb the TNMs. Finally, the TNM-loaded PNMP-AZ-FA was added to 10 mL of methanol to desorb the TNMs from the nanoparticles, followed by washing three times with methanol (2 mL each time) to ensure that all TNMs captured by PNMP-AZ-FA were collected. The above adsorption–desorption steps were repeated three times. After the last adsorption, the purple fermentation extract solution turned pale yellow, indicating that all TNMs had been completely adsorbed and captured. The TNM-containing liquids collected from the three capture cycles were concentrated using a vacuum rotary evaporator, and the residue was dissolved in 5 mL of chromatographic-grade methanol. The sample was then analyzed by UPLC-HRMS under the following conditions. Chromatographic separation was performed on an ACQUITY UPLC^®^ BEH C18 column (2.1 mm × 100 mm, 1.7 μm) maintained at 40 °C. The mobile phase consisted of water containing 0.1% (*v*/*v*) formic acid (A) and acetonitrile containing 0.1% (*v*/*v*) formic acid (B), with a flow rate of 0.3 mL/min and an injection volume of 2 μL. The gradient elution program was as follows: 0–3 min, 95% A isocratic; 3–18 min, linear decrease from 95% A to 5% A; 18–21 min, 5% A isocratic; 21–22 min, linear increase from 5% A to 95% A; and 22–25 min, re-equilibration at 95% A. Mass spectrometry was performed on an electrospray ionization (ESI) source in negative-ion mode, with a scan range of m/z 250–500. The heating block and curved desolvation line (CDL) temperatures were both set at 200 °C. The nebulizing gas flow was 1.5 L/min, and the drying gas pressure was 186 kPa. The ion trap (IT) and time-of-flight (TOF) vacuum pressures were 3.7 × 10^−3^ Pa and 8.5 × 10^−5^ Pa, respectively. The ion spray voltage was −3.5 kV. The recycling capacity of PNMP-AZ-FA for TNM A and TNM D was evaluated next. Briefly, 5 mL of methanol containing 3 mg of TNM A or TNM D was added to 45 mL of an aqueous dispersion containing 50 mg of PNMP-AZ-FA at room temperature. The mixture was stirred in a shaker for 5 min, and the PNMP-AZ-FA loaded with TNM A or TNM D was separated from the mixture via a magnet. Then the TNMs were desorbed from the NPs with 5 mL of methanol three times. The adsorption capacity was defined as the ratio of the adsorbed TNMs to the added TNMs (3 mg each). In the recycling test, the adsorption efficiency for each cycle was calculated as the ratio of the amount of TNM A or TNM D obtained after thorough elution with methanol from the PNMP-AZ-FA-captured nanoparticles to the initial added amount (3 mg). The TNM-desorbed PNMP-AZ-FA NPs were re-dispersed into 45 mL of water to start a new cycle of TNM adsorption and desorption. The recycling performance of the PNMP-AZ-FA matrix for the TNMs was evaluated for 20 cycles. For each cycle, three independent duplicates were analyzed.

### 3.7. Characterization of the NPs

The synthesized Fe_3_O_4_, Fe_3_O_4_@SiO_2_, and PNMP-AZ-FA were characterized by multiple spectroscopic techniques. Transmission electron microscopy (TEM) was employed to characterize the morphology of these materials (FEI Tecnai G2, USA). The X-ray diffraction (XRD) data were obtained on a D8 ADVANCE instrument (Bruker, Berlin, Germany) in the reflection mode using a Cu target at 40 kV and 30 mA to identify the phase and crystallite size of these NPs. The Fourier transform infrared spectroscopy (FTIR) measurements were performed at room temperature using the KBr tablet method on a Nicolet iS10 FTIR spectrometer (Thermo Scientific, Waltham, MA, USA). The zeta potential data were obtained using a Mastersizer 2000 (Malvern, UK). The saturation magnetization data were obtained (at 300 K) on a vibrating sample magnetometer (Quantum Design, San Diego, CA, USA), and the data were acquired to evaluate the magnetic properties of Fe_3_O_4_, Fe_3_O_4_@SiO_2_, and PNMP-AZ-FA.

### 3.8. Loading Capacity and Structural Stability Test

The loading capacity of the synthesized PNMP-AZ-FA was evaluated by adding 0.8 g of PNMP-AZ-FA and different amounts of TNM A or TNM D to 50 mL of 10% methanol solution. After incubation for 5 min, the NPs were separated using a magnet and then washed with 5 mL of 10% methanol three times. The TNMs adsorbed to the NPs were eluted using 5 mL of methanol three times, and the eluted TNMs were collected for HPLC (Waters Corporation, Milford, MA, USA) analysis to calculate the loading capacity.

Because the enediyne natural products are labile in acidic and reductive environments, the influence of the PNMP-AZ-FA on the stability of TNM A and TNM D during the adsorption process was tested. Firstly, 0.2 mg of TNM A or TNM D was dissolved in 20 mL of methanol. Then 5 mg of PNMP-AZ-FA was added to 80 mL of deionized water and dispersed by sonication for 20 min. After combining the methanol solution containing TNMs and water with PNMP-AZ-FA, the mixture was stirred for 5 min, and the magnetic particles were collected with a magnet; then the particles were stored at room temperature for 12 h. TNM A or TNM D was eluted from the surface of PNMP-AZ-FA using methanol three times (10 mL each time) to make sure all the TNMs were collected. The solvents were removed in a rotary evaporator. The TNM A or TNM D sample was redissolved in 2 mL of methanol and analyzed by HPLC and LCMS-IT-TOF mass spectrometer (Shimadzu Corporation, Kyoto, Japan). The HPLC and HPLC-MS conditions for TNM A and TNM D were the same as the detection conditions for AZ, except that the detection wavelengths were changed to 254 nm and 540 nm.

### 3.9. FR Expression Levels in Different Cells and the Antitumor Activity of PNMP-AZ-FA@TNMA

Cryopreserved HeLa, H9C2, KB, 4T1, MCF7, NIH-3T3, and SKOV3 cells obtained from the National Collection of Authenticated Cell Cultures (Shanghai, China) were resuscitated and seeded into culture dishes containing DMEM (Thermo Fisher Scientific, Waltham, MA, USA) medium, followed by incubation at 37 C in a 5% CO_2_ incubator. After the cells adhered and grew to confluence, they were subcultured. The cells were collected, washed three times with pre-cooled PBS, and lysed on ice for 5 min with 200 µL of lysis buffer. The lysate was collected and centrifuged at 12,000× *g* for 10 min at 4 °C and the supernatant was harvested. Protein concentration was determined using a BCA kit (Thermo Fisher Scientific, Waltham, MA, USA), and protein levels in each sample were normalized. The normalized lysates were mixed with loading buffer at a ratio of 4:1, denatured by boiling at 100 C for 5 min, cooled, and then separated by 10% SDS-PAGE. Subsequently, the proteins were electro-transferred onto a methanol-activated PVDF membrane (250 mA, 90 min). The membrane was blocked with 5% skim milk and incubated with Anti-β-actin primary antibody (monoclonal, Catalog: AF7018, Affinity Biosciences, Cincinnati, OH, USA; 1:2000 dilution) and FR primary antibody (monoclonal, Catalog: DF4058, Affinity Biosciences, Cincinnati, OH, USA; 1:1000 dilution) at 37 C for 2 h. After five washes with TBST, the membrane was incubated with secondary antibody (1:500 dilution) at 37 C for 2 h, followed by another washing step. Immunodetection was performed using an ECL chemiluminescence reagent, and the bands were imaged and quantitatively analyzed using ImageJ v1.54g (National Institutes of Health, Bethesda, MD, USA).

Separately, cells in the logarithmic growth phase were trypsinized, counted, and seeded into 96-well plates at a density of 3 × 10^4^ cells/mL (100 µL/well). After 24 h of culture, the medium was removed and replaced with fresh medium containing the corresponding drugs. Following 72 h of incubation, the medium was discarded, and the cells were gently washed with PBS. Subsequently, 100 µL of MTT solution (1 mg/mL; Sigma-Aldrich, St. Louis, MO, USA) was added to each well. After 4 h of incubation, the MTT solution was removed, and 100 µL of DMSO was added to each well. The plates were shaken for 1 min to mix, and the absorbance of each well was measured at 492 nm using a microplate reader. All in vitro experiments were performed with three independent biological replicates, and each biological replicate included three technical replicates unless otherwise stated. Data are presented as mean ± SD. Statistical analysis was performed using GraphPad Prism 9.0 (San Diego, CA, USA). Differences among multiple groups were analyzed by one-way analysis of variance (ANOVA) followed by Tukey’s multiple-comparison test. A value of *p* < 0.05 was considered statistically significant.

### 3.10. In Vivo Antitumor Evaluation

Male BALB/c nude mice (6 weeks old, 18–22 g) bearing tumors with a volume of approximately 200 mm^3^ were randomly divided into four groups (*n* = 6 per group), including the PNMP-AZ-FA group, the TNM A group, the PNMP-AZ@TNM A group, and the PNMP-AZ-FA@TNM A group. The TNM A-containing formulations were prepared at a concentration of 10 nM. Mice in each group were administered the corresponding formulation by tail-vein injection at 100 µL per injection once every two days, for a total of three injections. The animal experiments were approved by the Laboratory Animal Ethics Committee of Tianjin University of Traditional Chinese Medicine (approval No. TCM-LAEC2025541W2145). During the administration period, tumor volume and body weight were measured and recorded every 48 h. Tumor volume was calculated as V = length × width^2^/2. At 24 h after the final administration, mice were anesthetized by intraperitoneal injection of sodium pentobarbital (50 mg/kg) and subsequently euthanized by cervical dislocation. Tumors and major organs (heart, liver, spleen, lungs, and kidneys) were completely excised. Tissue samples were fixed and subjected to hematoxylin and eosin (H&E) staining for histopathological examination. Statistical analysis: All data are presented as mean ± SEM. Tumor volumes measured repeatedly over the treatment period were analyzed using two-way repeated-measures analysis of variance (ANOVA), with treatment group and time as factors, followed by Bonferroni’s post-hoc test for multiple comparisons among the four groups at each time point. Final weights were compared using one-way ANOVA with Bonferroni’s post-hoc test. Differences were considered statistically significant when *p* < 0.05. All statistical analyses were performed using GraphPad Prism 9.0 (GraphPad Software, San Diego, CA, USA).

## 4. Conclusions

The synthesized AZ-modified nanomagnetic particles PNMP-AZ-FA showed high loading capacity, quick separation and adsorption/desorption, and good recyclability for the separation and extraction of TNMs. The feasibility was analyzed by docking AZ with TNMs and constructing PEI-AZ/TNMs dynamic simulation models. The suitable adsorption systems (in 10% methanol) were confirmed via theoretical simulation and experimental verification. Compared with the traditional methods, this study provides an efficient, economical, and environmentally friendly separation process for anthraquinones. The cost comparison should be interpreted as a direct reagent-cost estimate rather than a complete process-cost analysis. The results demonstrate the application prospects of functionalized magnetic NPs in the target-specific separation of compounds from plant and microbial crude extracts. Furthermore, the FR-targeted nanomaterial PNMP-AZ-FA constructed in this study leverages FA to specifically anchor to FR overexpressed on tumor cell surfaces, while its AZ-modified magnetic core efficiently adsorbs anthraquinone drugs, ultimately achieving tumor-targeted drug delivery. Compared with the non-targeted material PNMP-AZ, this folate-modified system exhibited improved tumor inhibition effects in the FR-high KB model under the tested conditions. However, the current biological data do not directly prove receptor-dependent uptake, increased tumor accumulation, reduced systemic exposure, or long-term safety. Future work should include AZ-free adsorption controls, quantitative recovery/enrichment analysis, hydrodynamic and serum-stability characterization, receptor-blocking or folate-competition experiments, biodistribution, pharmacokinetic, hematological, and biochemical studies. In conclusion, this study validates the bifunctional design of engineered magnetic nanoparticles (MNPs) primarily for TNM capture and recovery and provides preliminary evidence for a folate-associated delivery application.

## Data Availability

The original contributions presented in this study are included in the article/supplementary material. Further inquiries can be directed to the corresponding authors.

## Supplementary Materials

The following supporting information can be downloaded at: https://www.mdpi.com/article/10.3390/molecules31193531/s1. Table S1: The interaction energy of TNMs and AZ; Table S2: The cost of organic reagents required to obtain TNMs from crude fermentation mixture by traditional methods and PNMP-AZ-FA method; Table S3: NMR chemical shift data for AZ-ECH (in [D6] DMSO, 298K); Table S4: Characterization data of Fe_3_O_4_, Fe_3_O_4_@SiO_2_, and PNMP-AZ-FA; Figure S1: 2D Chemical structure of PEI-AZ; Figure S2: Molecular dynamic simulation for the process of PEI-AZ/TNM A adsorption/desorption in pre-set systems with different ratio of methanol/water spheres. 0%, 5%, 10%, 20%, 30%, 40%, 100% represent different methanol concentration in the spheres, respectively. The binding of TNM A to PEI-AZ increased first and then weakened with the increase of methanol concentration, and 10% of methanol show the strongest binding performance, and almost all TNM A molecules are bound to the surface of PEI-AZ in 20 ns; Figure S3: Molecular dynamic simulation for the process of PEI-AZ/TNM D adsorption/desorption in pre-set systems with different ratio of methanol/water spheres. 0%, 5%, 10%, 20%, 30%, 40%, 100% represent different methanol concentration in the spheres, respectively. The binding of TNM D to PEI-AZ increased first and then weakened with the increase of methanol concentration, and 5%, 10% of methanol show the strongest binding performance, and almost all TNM D molecules are bound to the surface of PEI-AZ in 20 ns; Figure S4: RMSD of (**A**) PEI-AZ/TNM A and (**B**) PEI-AZ/TNM D; Figure S5: HPLC analysis of the elements obtained by PNMP-AZ-FA in fermentation products of Streptomyces sp. CB03234-S; Figure S6: Full wavelength scan analysis of the compounds captured by PNMP-AZ-FA at different time in HPLC; Figure S7: The structural-stability performance of TNMs. HPLC analysis results of the structural-stability of (**a**) TNM A and (**b**) TNM D; HPLC-MS analysis results of the structural-stability of (**c**)TNM A and (**d**) TNM D; Figure S8: Evaluation of the antitumor efficacy of different materials against H9C2 cells; Figure S9: Gross appearance of KB tumor xenografts in nude mice following 14 days of drug treatment; Figure S10: Tumor morphology and volume statistics of harvested tumors from 4T1 tumor-bearing mice after 2 weeks of treatment with different drugs. Reference [46] is cited in the Supplementary Materials.
