# Supplementary material for: A Dual-Functional Magnetic Nanoparticle for Rapid Extraction and Targeted Tumor Delivery of Tiancimycin
Source: Molecules. 2026 Oct 3;31(19):3531. doi: 10.3390/molecules31193531 (PMC13666505; doi:10.3390/molecules31193531)
Supplement: Supplementary file 1 [file molecules-31-03531-s001.zip › molecules-4474499-supplementary.pdf]

Table S1 The interaction energy of TNMs and AZ.

Sample	AZ/TNM A (Hartree)	AZ/TNM D (Hartree)
Complex	−2536.693	−2879.206
AZ	−839.488	−839.490
TNMs	−1697.177	−2039.682
Binding energy	−0.027 (−71.765 kJ)	−0.033 (−88.113 kJ)

Table S2 The cost of organic reagents required to obtain TNMs from crude fermentation mixture by traditional methods and PNMP-AZ-FA method.

			**Traditional method**		**New method**	
Component	Supplier	Catalog Number	Quantity	Price ($)	Quantity	Price ($)
MeOH	Concord	8001LC6030	5L	35.7	200 mL	1.4
EtOAc	Adamas	23272Q-ZX	2L	43.2	0 mL	0
TFA	TCI	T0431-25G	4 mL	1.5	0 mL	0
Fe_3O_4@SiO_2@PEI-AZ	Made by our lab	--	0g	0	0.4g	0.7
MCI gel	Mitsubishi Chemical Holdings	7D831	50g	115.3	0g	0
Sehadex LH-20 gel	GE Healthcave	17-0090-2	30g	150.3	0g	0
Total				346.0		2.1

The crude extract of TNMs was obtained from 7.5 L of fermentation product.

Table S3 NMR chemical shift data for AZ-ECH (in [D6] DMSO, 298K)

Pos.	AZ (in *d*6-DMSO)[1]		AZ-ECH (in *d*6-DMSO)				
	δ_C	δ_H	δ_C	δ_H, muti, (*J* in Hz)	COSY	HMBC	DEPT
1	154.2	/	152.36	/	/	7.45, 12.72	/
2	152.28	/	153.01	/	/	4.02, 4.53, 7.71, 12.72	/
3	122.70	7.27	118.56	7.45	7.71	7.71	↑
4	117.73	7.70	120.90	7.71	7.45	/	↑
4a	122.31	/	125.50	/	/	7.45	/
9a	125.26	/	116.38	/	/	7.71, 12.72	/
5	128.59	8.23	127.27	8.16	7.92	7.92	↑
6	135.04	7.95	135.69	7.92	8.16, 8.22	8.22	↑
7	134.16	7.95	134.74	7.92	8.16, 8.22	8.16	↑
8	127.95	8.23	127.05	8.22	7.92	7.92	↑
8a	135.51	/	133.35	/	/	7.92	/
10a	136.57	/	133.90	/	/	7.92	/
9	190.25	/	189.11	/	/	7.71, 8.22	/
10	182.03	/	181.20	/	/	7.71, 8.16	/
11	/	/	70.61	4.02	3.42, 4.53	2.77, 2.90	↓
				4.53	4.02		
12	/	/	49.88	3.42	4.02	2.77, 2.90, 4.02, 4.53	↑
13	/	/	44.24	2.77	2.90	4.02, 4.53	↓
				2.90	2.77		
14	/	/	/	12.72	/	/	/
2-OH	/	8.23	/	/	/	/	/

Table S4 Characterization data of Fe_3O_4, Fe_3O_4@SiO_2, and PNMP-AZ-FA.

	ζ-potential （mV）	Average diameter	loading efficiency (%)	
			TNMA	TNMD
Fe_3O_4	-16.7±1.3	156.3±17.3	9.3±2.1	9.8±2.6
Fe_3O_4@SiO_2	-27.8±2.1	184.5±21.5	8.4±1.9	9.1±2.4
PNMP-AZ-FA	-35.3±2.9	202.3±26.7	98.9±2.8	99.5±1.1

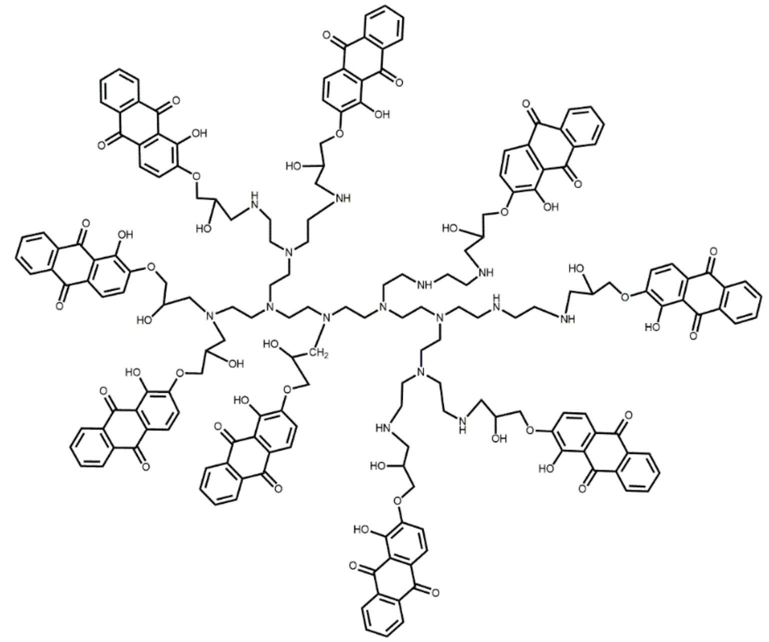

Figure S1 2D Chemical structure of PEI-AZ.

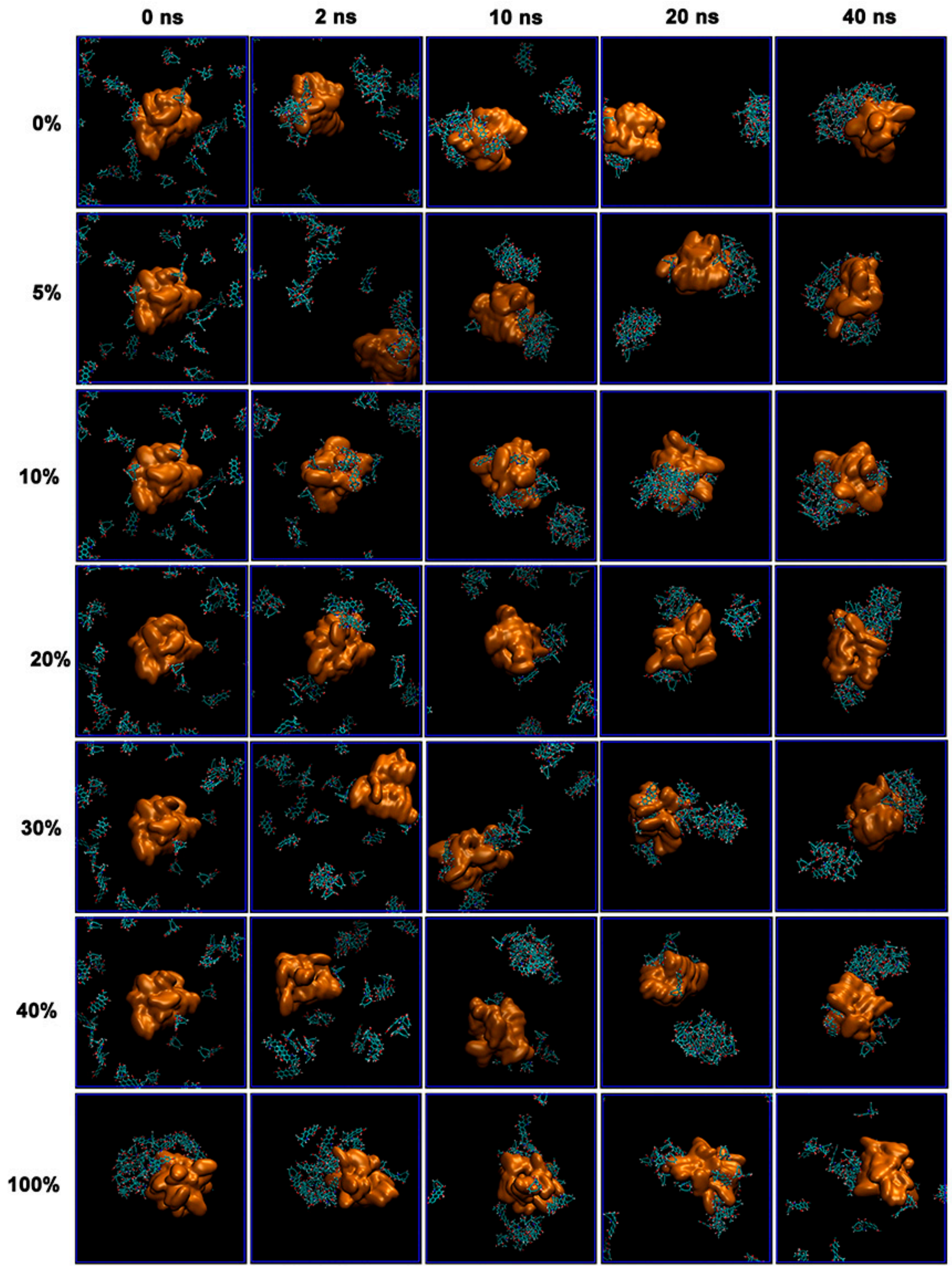

Figure S2 Molecular dynamic simulation for the process of PEI-AZ/TNM A adsorption/desorption in pre-set systems with different ratio of methanol/water spheres. 0%, 5%, 10%, 20%, 30%, 40%, 100% represent different methanol concentration in the spheres, respectively. The binding of TNM A to PEI-AZ increased first and then weakened with the increase of methanol concentration, and 10% of methanol show the strongest binding performance, and almost all TNM A molecules are bound to the surface of PEI-AZ in 20 ns.

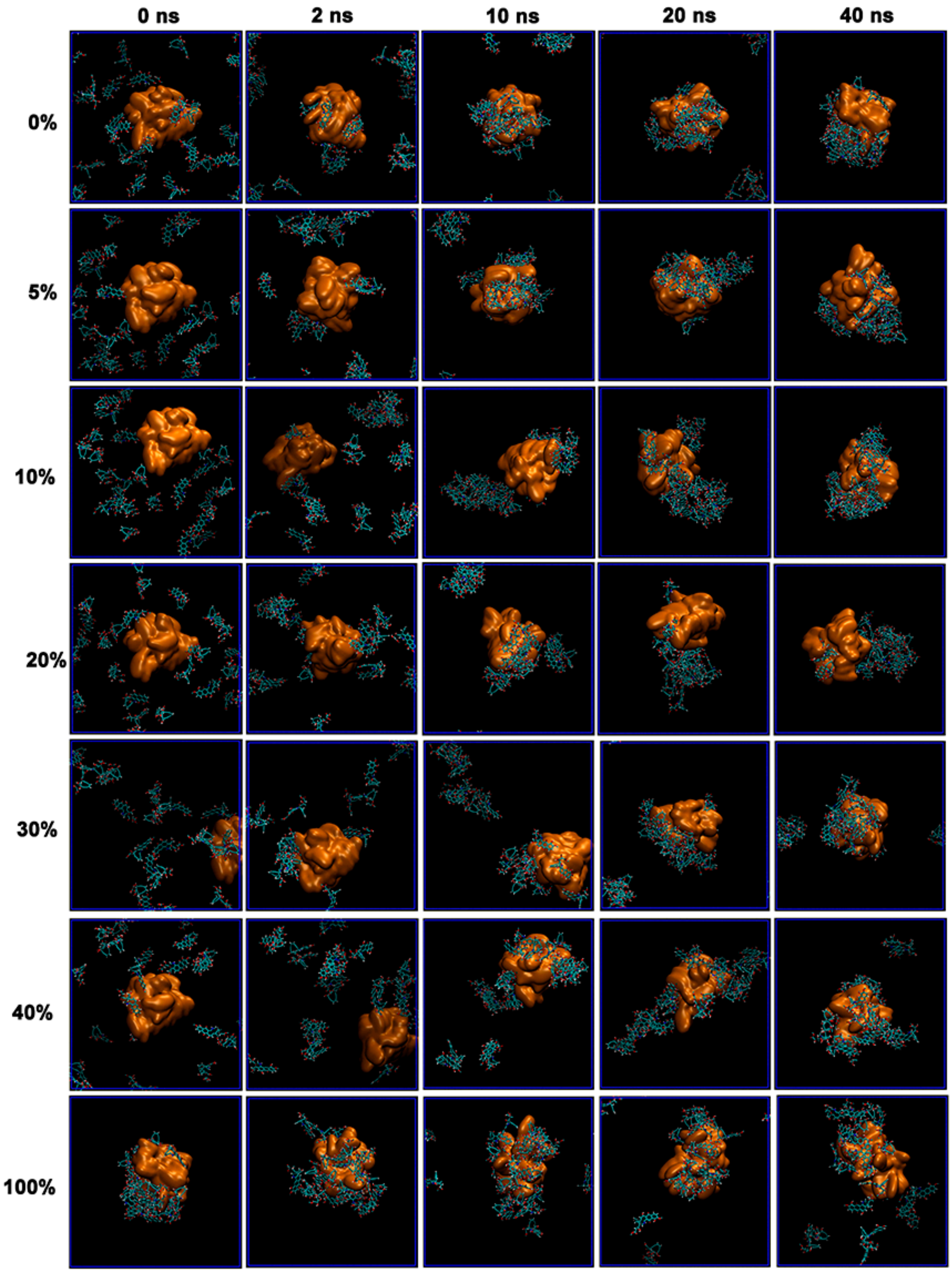

Figure S3 Molecular dynamic simulation for the process of PEI-AZ/TNM D adsorption/desorption in pre-set systems with different ratio of methanol/water spheres. 0%, 5%, 10%, 20%, 30%, 40%, 100% represent different methanol concentration in the spheres, respectively. The binding of TNM D to PEI-AZ increased first and then weakened with the increase of methanol concentration, and 5%, 10% of methanol show the strongest binding performance, and almost all TNM D molecules are bound to the surface of PEI-AZ in 20 ns.

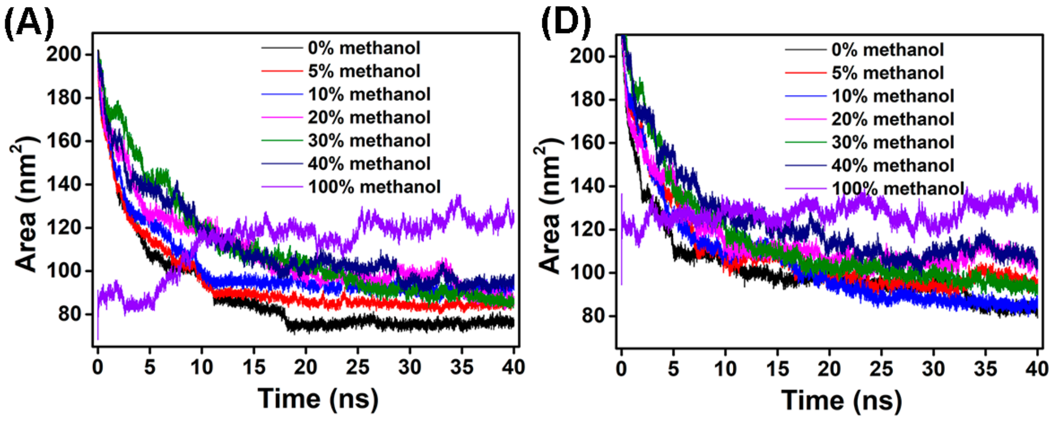

Figure S4 RMSD of A) PEI-AZ/TNM A and B) PEI-AZ/TNM D.

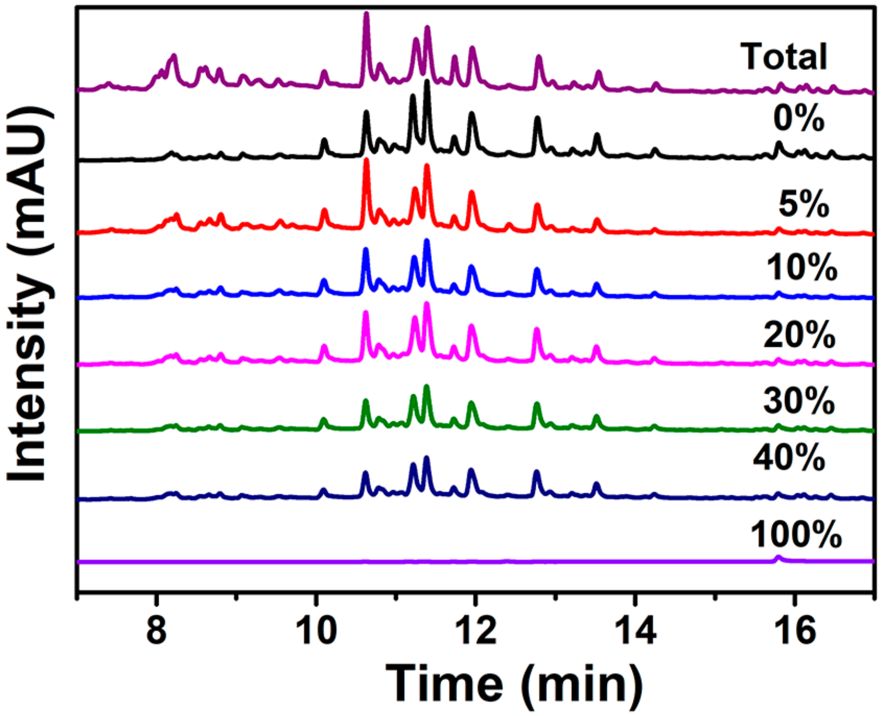

Figure S5 HPLC analysis of the elements obtained by PNMP-AZ-FA in fermentation products of *Streptomyces sp.* CB03234-S.

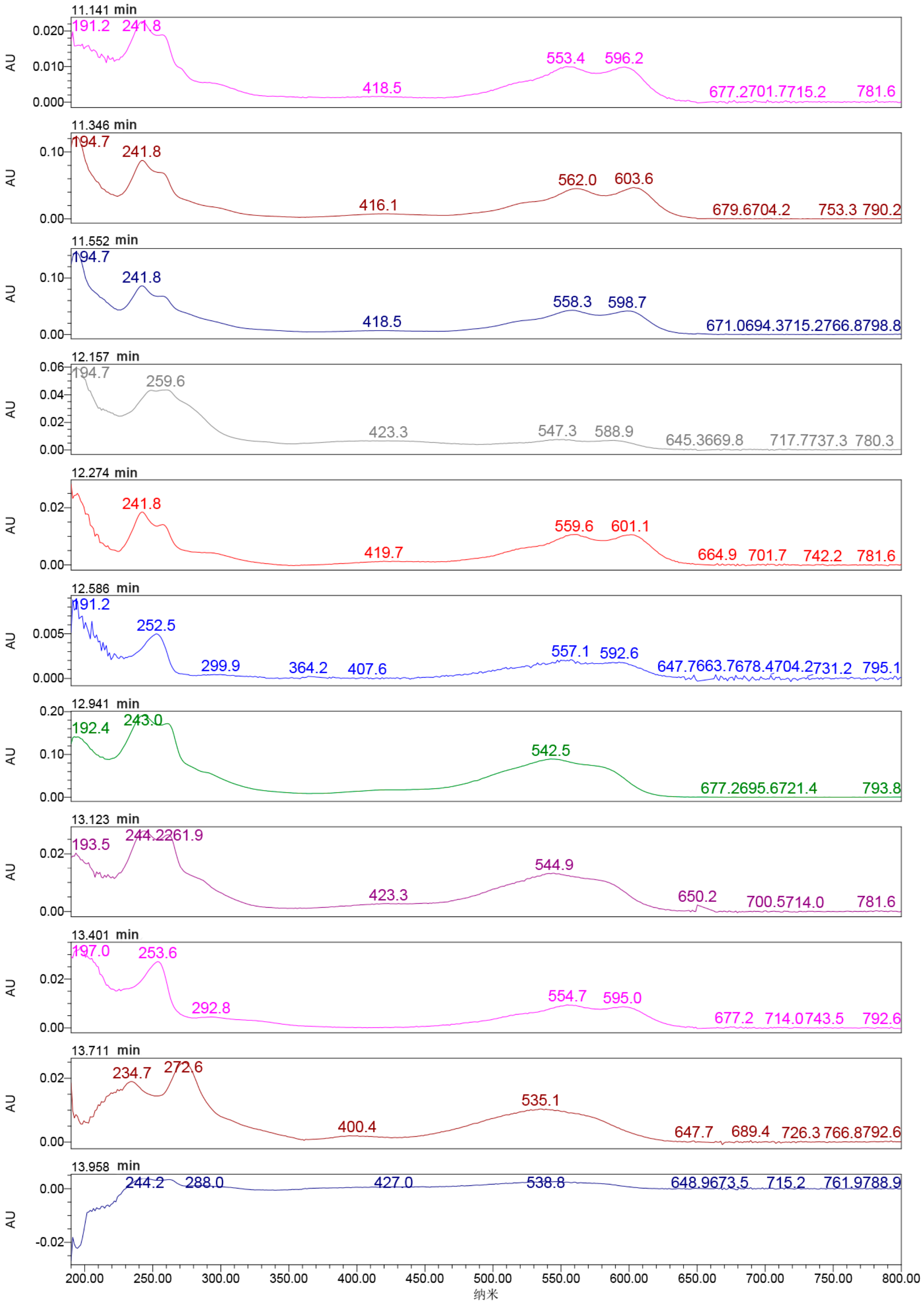

Figure S6 Full wavelength scan analysis of the compounds captured by PNMP-AZ-FA at different time in HPLC.

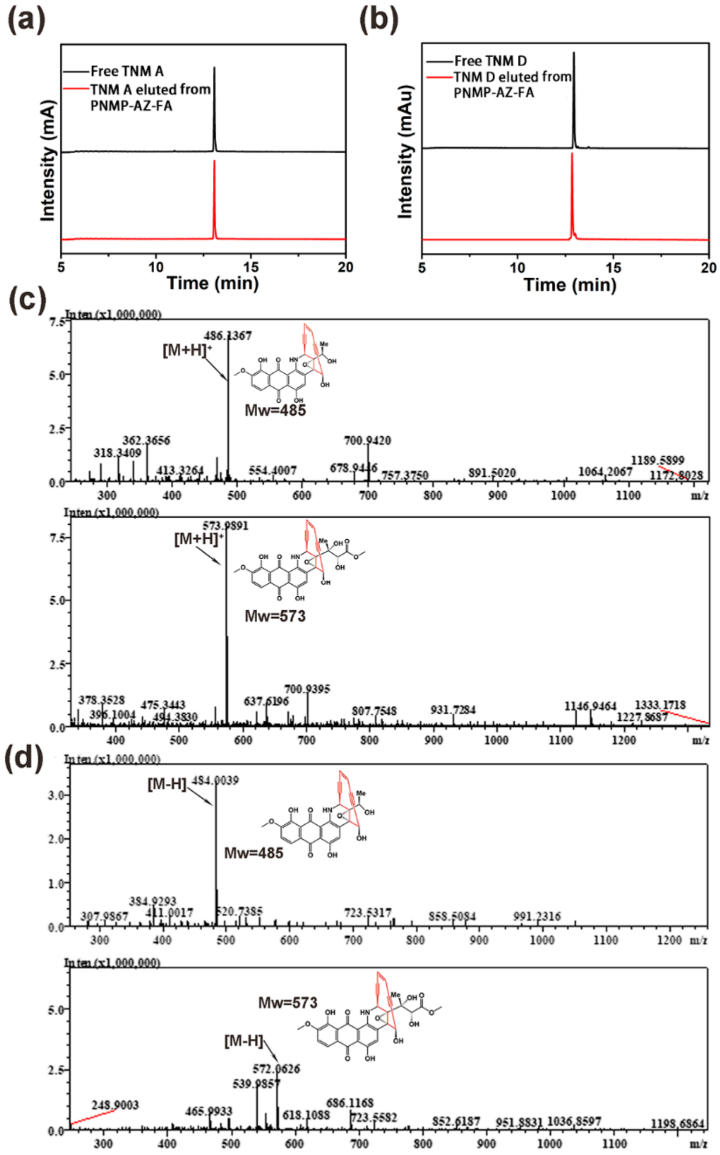

Figure S7 The structural-stability performance of TNMs. HPLC analysis results of the structural-stability of (a) TNM A and (b) TNM D; HPLC-MS analysis results of the structural-stability of (c)TNM A and (d) TNM D.

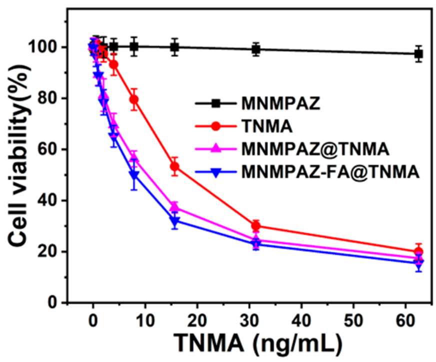

Figure S8 Evaluation of the antitumor efficacy of different materials against H9C2 cells.

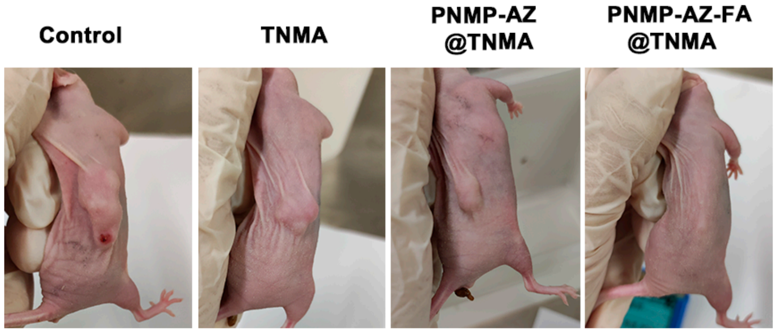

Figure S9 Gross appearance of KB tumor xenografts in nude mice following 14 days of drug treatment.

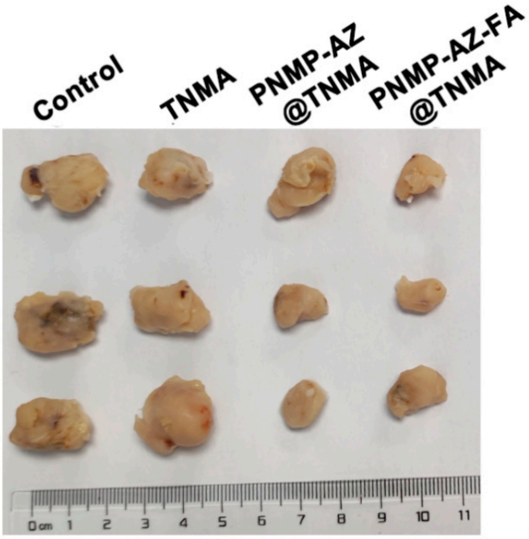

Figure S10 Tumor morphology and volume statistics of harvested tumors from 4T1 tumor-bearing mice after 2 weeks of treatment with different drugs.

[1] Dhananjeyan M R, Milev Y P, Kron M A, Nair M G. Synthesis and activity of substituted anthraquinones against a human filarial parasite, Brugia malayi. Journal of Medicinal Chemistry, 2005, 48(8): 2822-2830.
